# Pleiotropic Role of HSF1 in Neoplastic Transformation

**DOI:** 10.2174/1568009614666140122155942

**Published:** 2014-02

**Authors:** Natalia Vydra, Agnieszka Toma, Wieslawa Widlak

**Affiliations:** Maria Sklodowska-Curie Memorial Cancer Center and Institute of Oncology, Gliwice Branch, Wybrzeze Armii Krajowej 15, 44-101 Gliwice, Poland

**Keywords:** cancer, drug resistance, genomic instability, HSF1 inhibitors, HSPs, metastasis, p53 signaling

## Abstract

HSF1 (Heat Shock transcription Factor 1) is the main transcription factor activated in response to proteotoxic stress. Once activated, it induces an expression of heat shock proteins (HSPs) which enables cells to survive in suboptimal conditions. HSF1 could be also activated by altered kinase signaling characteristic for cancer cells, which is a probable reason for its high activity found in a broad range of tumors. There is rapidly growing evidence that HSF1 supports tumor initiation and growth, as well as metastasis and angiogenesis. It also modulates the sensitivity of cancer cells to therapy. Functions of HSF1 in cancer are connected with HSPs’ activity, which generally protects cells from apoptosis, but also are independent of its classical targets. HSF1-dependent regulation of non-HSPs genes plays a role in cell cycle progression, glucose metabolism, autophagy and drug efflux. HSF1 affects the key cell-survival and regulatory pathways, including p53, RAS/MAPK, cAMP/PKA, mTOR and insulin signaling. Although the exact mechanism of HSF1 action is still somewhat obscure, HSF1 is becoming an attractive target in anticancer therapies, whose inhibition could enhance the effects of other treatments.

## INTRODUCTION

All organisms are able to respond to heat shock by specific changes in the pattern of gene expression, leading to an elevated synthesis of heat shock proteins (HSPs). HSPs are major molecular chaperones, which assist protein folding during synthesis and repair, or contribute to degradation under proteotoxic stress. Mammalian HSPs are classified according to molecular weight into several families: HSPH (HSP110), HSPC (HSP90), HSPA (HSP70), DNAJ (HSP40), and HSPB (small HSPs, sHSPs). Each family includes members that are constitutively expressed, strictly inducible by stress, and/or targeted to different cellular compartments [1]. 

Expression of *HSP* genes is regulated by heat shock factors (HSFs), which are a structurally and functionally conserved class of proteins. A single *HSF* gene has been isolated from *Saccharomyces cerevisiae* and *Drosophila melanogaster*. Several members of the HSF family have been found in vertebrates (HSF1 through HSF5, HSFY, HSFX; see Table **1**) and plants (HSFA1, HSFA2, HSFB1 and others). Expression of *HSF* genes in most species is constitutive and not stress-responsive. Among vertebrates, HSF1, HSF2, and HSF4 are ubiquitous, whereas HSF3 has been described only in birds and mice. Each of them exists in two isoforms generated by alternative splicing. HSF1 is the functional homolog of the HSF found in lower eukaryotes. It is activated *in vivo* by heat shock and numerous forms of physiological stress. HSF3 functions as a heat-responsive factor, exhibiting a delay of heat-shocked activation. In contrast to HSF1 and HSF3, HSF2 is not activated in response to stress stimuli (although it can actively modulate the heat shock response), but under developmentally related conditions. HSF4 is expressed in a tissue-specific manner and displays constitutive DNA-binding activity [2]. According to data from the BioGPS gene annotation portal [3], *HSF5* is highly expressed almost exclusively in testes, yet its function and characteristics remain to be elucidated. Poorly characterized *HSFY* and *HSFX* genes both exist in two identical copies on the Y or X chromosome, respectively [4]. Although deletion of the Y chromosome region containing the *HSFY* is associated with azospermia and deteriorated spermato-genesis, HSFY is not required for spermatocyte maturation [5]. 

HSF1 is a master regulator of the heat shock response, which is the major mechanism of cell adaptation to environmental stress. Moreover, HSF1 is involved in processes associated with development and growth [6, 7], fertility [8-10] and longevity in some organisms [11]. In addition, HSF1 is a potent factor supporting tumor growth. It has been shown that mouse embryonic fibroblasts (MEFs) with HSF1 knockout (*Hsf1-/-*) revealed a decreased ability to neoplastic transformation induced by the introduction of mutated HRAS (*V12D*), or overexpression of PDGFB (platelet-derived growth factor beta polypeptide). Furthermore, *Hsf1-/-* mice were less susceptible to chemically induced skin or liver carcinogenesis and to neoplastic transformation driven by expression of ERBB2/NEU (HER2) or mutant p53 [12-15]. Down-regulation of HSF1 expression by siRNA also had a great impact on the viability of tumor cells, but it was neutral for non-tumor cell lines [12,  16].

HSF1 is overexpressed in a broad range of tumors and tumor cell lines. Its high nuclear accumulation was first detected in the metastatic PC-3M prostate cancer cell line [17]. A high HSF1 expression was also detected in colorectal cancer [18], breast cancer [19], oral squamous cell carcinoma [20], hepatocellular carcinoma [21], multiple myeloma [22], glioma [23], and ovarian tumors [24]. High nuclear accumulation of HSF1 was detected in xenograft tumors formed by highly malignant cells in immunocompromised mice, and also in human prostate, colon, lung, pancreas, and cervix carcinomas [16]. High HSF1 expression has been associated with a reduced survival rate of patients with breast cancer [25] and was proposed as an independent prognostic factor for overall survival in patients with hepatocellular carcinoma [21]. *HSF1* has also been identified as one of the only six potent metastasis-promoting genes in a genome-wide screen for enhancers of invasion by malignant melanoma cells [26]. HSF1 does not play a role of classical oncogene or tumor suppressor in carcinogenesis, but its activity influences many aspects of cell metabolism enabling tumor growth, which is summarized schematically in Fig. (**1**). Such a mechanism was called “non-oncogenic addiction” [27]. The essential role HSF1 in carcinogenesis makes it an attractive target for anti-cancer strategies. Here, we review the possible function of HSF1 in cancer biology.

## MECHANISM OF HSF1 ACTION AND ITS IMPACT ON GENE EXPRESSION

Under physiological conditions HSF1 exists as a monomer localized predominantly in the cytoplasm. The monomeric structure of HSF1 is stabilized by its interactions with chaperone protein complexes, mainly by HSP90s in complex with p23 protein and immunophilin. During stress conditions, when the level of unfolded proteins increases, HSPs are released from complexes with HSF1 and serve as molecular chaperones for unfolded molecules. An elevated level of unbound HSF1 promotes its oligomerization, translocation to nuclei and DNA binding [28]. This process is additionally regulated by elongation transcription factor (eEF1a) and non-coding RNA, HSR1 [29]. In turn, the release of HSF1 from DNA and its monomerization is possible due to interaction of its transactivation domain with HSPA, DNAJ, and HSBP1 proteins [30,  31]. HSP90-p23-immunophilin complex also interacts with the trimeric form of HSF1, leading to HSF1 monomerization [32]. 

The trimeric form of HSF1 binds to specific regions in the genome called Heat Shock Elements (HSEs). HSE consists of the 5-bp module [nGAAn] arranged as contiguous inverted repeats [nTTCn|nGAAn|nTTCn|nGAAn]. The structure of HSE can be slightly diverse in different genes. The number of 5-bp blocks can vary, ranging from 2 to 4. Moreover the nucleotide sequence of HSE can be different from the perfect canonical sequence. The traditional view of HSF1 signaling has been referred to as the regulation of *HSP* genes expression during the heat shock response. Promoters of *HSP *genes contain at least three HSE elements located approximately 40 to 270 bp upstream of the transcriptional start site [2]. However, the human genome contains approximately 10,000-20,000 copies of a perfect consensus located in promoters, intragenic and intergenic regions. Studies of HSF1 binding to DNA on a genome-wide scale suggest that HSF1 may regulate genes involved in diverse cellular processes that extend far beyond protein folding (which is the general role of HSPs). In mammalian cells these processes include among others apoptosis, RNA splicing, and ubiquitination [33]. Studies on yeast and *Drosophila* revealed that HSF could be additionally involved in the regulation of carbohydrate metabolism, transport and cytoskeleton organization [34,  35]. 

Genome-wide analysis of HSF1 binding to gene promoter coupled with analysis of gene expression during heat shock revealed that HSF1 binding by itself does not confer heat-inducibility of a target gene [33,  36]. The balance between phosphorylation and dephosphorylation is an additional factor affecting HSF1 conformation and activity. Phosphorylation of several serine residues like Ser121 (by MK2 kinase) [37], Ser303 (by GSK3) [38], Ser307 (by ERK) [39] and Ser363 (by JNK/SAPK or PKC) [40] represses the HSF1 transcriptional activity. On the other hand, phosphorylation of Ser230 (by CaMKII) [41], Ser320 (by PKA) [42], Ser326 (by mTORC1) [43] and Thr142 (by CK2) [44] during stress conditions is essential for the transcriptional activity of HSF1. Phosphorylation of Ser419 by PLK1 plays a very important role in the accumulation of HSF1 in the nucleus [45]. Other post-translational modifications (like sumoylation, acetylation) are also involved in HSF1 activation and function [46]. 

It has been shown that in addition to environmental stress, HSF1 can be activated by other mechanisms, which is summarized schematically in Fig. (**2**). A simple loss of the NF1 tumor suppressor followed by elevated RAS/MAPK signaling leads to HSF1 activation *via *phosphorylation of Ser326 [23]. Such activation precedes malignancy. This mode of HSF1 activation allows cells to withstand a range of proteotoxic insults, even during the very early stages of carcinogenesis, thereby supporting the process. Ser326 of HSF1 has recently been found to be phosphorylated by mTORC1 [43], which can be a downstream target of signaling *via *RAS/RAF/MEK/ERK as well as *via *RAS/PI3K/PTEN/AKT/mTOR [47]. Thus, it seems that disturbances in these pathways or in any other which change the balance between the phosphorylation and dephosphorylation of HSF1, may activate this transcription factor. Indeed, it has been found that RET stimulation (which can transduce signaling through PI3K/AKT, PLCG1/G2, RAS/ERK, MAPK, and JNK pathways), leads to increased HSF1 activity [48]. HSF1 becomes transcriptionally active also following exposure of cells to heregulin (NRG1, neuregulin 1). Heregulin (the ligand of the EGFR family of receptors) triggers intracellular signaling cascades through PI3K/AKT1 (but not ERK), resulting in the inhibition of glycogen synthase kinase 3 (GSK3), which is HSF1 antagonist [49]. Consistently, inhibition of the PI3K/AKT/GSK3β pathway causes decreased expression of HSF1 and down-regulation of HSPs [50]. 

Overexpression of HSPs, which are the major targets of HSF1, was found in some tumors and in numerous cancer cell lines grown *in vitro* [51]. Thus, it has been assumed that HSF1 affects tumor initiation by regulation of HSPs expression, which also have an important role in cancer [52]. However, it seems that further tumor progression does not depend on HSPs and a distinct HSF1-regulated transcriptional program is realized in cancer cells. Activity of HSF1 correlates with tumorigenic potential of cells. In highly tumorigenic cells HSF1 regulates several cancer-specific genes, which are different from genes activated during the heat shock response. The involvement of HSF1 in the regulation of cell cycle, apoptosis, energy metabolism, adhesion and other processes was observed in malignant cell lines, but also in breast, colon, and lung tumors isolated directly from human patients. Based on this observation, the HSF1-cancer signature of 456 genes was established and its correlation with poor outcomes in diverse human cancers was determined [16]. 

In addition to its role as a transcription factor, HSF1 affects diverse cellular processes *via* its interactions with other proteins. HSF1 interacts with more than 90 different proteins (complete list available on the website http://www.ncbi.nlm.nih.gov/gene/3297, in bookmark *Interactions*), which interfere e.g. with chromatin remodeling, polyadenylation efficiency or mRNA transport into the cytoplasm.

## CROSS-TALK BETWEEN HSF1 AND P53 SIGNALING

It has been reported that HSF1 stimulates different cellular processes characteristic for tumor progression, including aneuploidy [53], anchorage-independent and mitogen-independent growth [12, 49], cell migration [54], angiogenesis [14,  55], chemoresistance, and autophagy [56]. Notably, HSF1 signaling is frequently dependent on p53 status, and both factors apparently interfere with each other.

p53 protein is a transcription factor, which regulates gene expression primarily in response to DNA damage, and activates either cell cycle arrest enabling DNA repair, or apoptosis (or senescence) if the DNA damage exceeds a certain threshold [57]. Because of its role in preventing genetic instability and mutation, p53 is known as “the guardian of the genome”. Proper function of p53 is supported by HSF1 in several different ways. First, HSF1 is required for the nuclear translocation of p53 [58]. The intracellular transport of p53 is conducted by microtubules of the cytoskeleton, which polymerization is dependent on different HSPs regulated by HSF1 [59]. Additionally, HSF1 enhances p53-mediated transcription in response to genotoxic stress, leading to cell cycle arrest in G2/M phase and apoptosis. Both p53 and HSF1 are recruited to certain p53-responsive genes (e.g. *CDKN1A* coding for p21 protein), and activate them. HSF1 is also necessary for the phosphorylation and the activation of p53 by ATR and CHK1 kinases in response to DNA damage [60]. On the other hand, when senescence is induced in response to DNA damage (*via *p53 and MAPK signaling), HSF1 is suppressed through the down-regulation of ELAVL1(HuR)/SIRT1 pathway, which in turn enhances the MAPK/NFκB signaling in a positive feedback loop, thus supporting chronic inflammation and senescence phenotype [61].

Inactivation of the proper p53 function due to mutations in the *TP53* gene leads to carcinogenesis. These mutations are detected in approximately 50% of malignant human tumors. Mutant p53 protein loses its tumor suppression activity and often gains additional oncogenic functions which endow cells with growth and survival advantages [62]. Notably, HSF1 can also support p53-mediated carcinogenesis. In mice carrying a clinically relevant hot spot mutation in the* Trp53 *gene (*R172H*; corresponding to codon 175 in human *TP53*), a broad spectrum of tumors is developed, which include osteosarcomas, hemangiosarcomas, B-cell lymphomas and a variety of carcinomas [63]. Development of these tumors is dependent on HSF1 and in *Hsf1* knockout mice tumor-free survival is dramatically prolonged [12]. p53 knockout mice (*Trp53*-/-) are also susceptible to spontaneous tumors, predominantly lymphomas [64]. Although an additional HSF1 deficiency does not prolong tumor-free survival, it shifts tumor development from lymphomas to testicular carcinomas and soft tissue sarcomas [65]. The selective suppression of lymphomas in mice deficient in *Trp53* and *Hsf1* is associated with an increased p53-independent apoptosis, altered cytokine signaling and suppressed production of inflammatory factors. It indicates a supportive role of HSF1 for tumor formation associated with loss of p53 function. Overexpression of HSF1 in cells without p53 leads to aneuploidy and genomic instability [66]. This links the supportive role of HSF1 for tumor formation (associated with the p53 loss) with regulation of mitotic cell cycle checkpoint. Interestingly, loss of the *Hsf4* gene, associated with induction of cellular senescence, also inhibits spontaneous tumorigenesis in mouse cancer models [67]. 

HSF1 apparently influences the stability of the p53 protein, however the exact mechanism remains unclear. Under physiological conditions the p53 protein level is regulated by the balance between its synthesis and degradation. Among cellular proteins affecting this balance is HSF1 and HSF1-regulated αB-crystallin (HSPB5). It has been shown that upon genotoxic stimulation *Hsf1*-/- MEFs accumulate p53 protein at significantly higher levels than the wild-type cells [68]. Stability of the mutant p53*R175H* protein (which is generally more stable than the wild-type p53) is also dependent on HSF1, yet different effects are observed. It has been shown that *Hsf1*-/- MEFs accumulate ectopic mutant p53 more efficiently than the wild type cells [68]. However, knockdown of HSF1 (or inhibition of HSF1-dependent HSP90) in human cancer cells results in the degradation of mutant p53 and increased cell mortality [69]. In both cases involvement of HSF1-dependent chaperones (small HSPs* vs *HSP90) and the HSP-dependent regulation of proteasomal degradation was suggested. It could be speculated that the observed discrepancy depends on the balance between chaperones in HSF1-deficient cells and their final impact on proteasome activity. The activity of HSF2, which is differentially expressed in both types of cells, could additionally contribute to the observed differences [70]. 

## HSF1 AND GENOMIC INSTABILITY

Tumor progression is accompanied by a wide range of genome instability, from chromosome rearrangement to aneuploidy, which are observed in virtually all cancers. In general, chromosomal instability arises as a consequence of DNA replication failure, centrosome-duplication failure or deregulation of mechanisms controlling the cell division, mainly the mitotic checkpoint [71]. It was suggested that HSF1 is required for proper mitotic progression, since disturbances in the end phase of mitosis have been observed in *Hsf1*-/- MEFs [72]. 

Active HSF1 directly regulates the expression of the *FOXM1* gene, which plays a key role in the cell cycle progression through G2/M [73]. Abnormal up-regulation of FOXM1, found in the majority of solid human cancers, correlates with an elevated expression of HSF1, which could directly induce genomic instability [73, 74]. However, HSF1 interferes with the cell cycle also due to its interactions with other proteins. In early mitosis HSF1 localizes to the centrosome and close to the kinetochore region of the mitotic chromosomes. It is phosphorylated at Ser216 by PLK1, which is a major mitotic regulator kinase. Phosphorylated HSF1 interacts with CDC20 protein (cell-division cycle protein 20). HSF1 is then ubiquitynated by the SCFβ-TrCP complex and degraded. Released CDC20 interacts with MAD2 (mitotic arrest deficient 2), and then with APC (anaphase promoting complex), which enables metaphase to anaphase transition. Consequently, *Hsf1*-/- MEFs do not show proper mitotic progression, which results in a high percentage of multinucleated cells [72]. On the other hand, over-phosphorylation of HSF1 also can inhibit mitotic exit, which results in aneuploidy and multinucleated cells [53]. HSF1-dependence of genomic instability was observed in many cancer cell lines with inactivated *TP53 *gene [66, 75]. In cells with mutant p53 protein, an excessive phosphorylation of HSF1 by PLK1 is observed, which stabilizes interactions of HSF1 with CDC20. In this case CDC20-MAD2 interactions are inhibited, which blocks metaphase to anaphase transition leading to aneuploidy [53,  66, 72]. 

## HSF1 AND REGULATION OF CANCER CELL METABOLISM 

An altered metabolism of cancer cells supports both growth factor-independent ribosome biogenesis, and synthesis of fatty acids and membrane lipids [76]. The uptake of nutrients, particularly glucose, is enhanced in cancer cells to meet or exceed the bioenergetic demands of their growth and proliferation [77]. Normal differentiated cells rely primarily on mitochondrial oxidative phosphorylation to generate the energy needed for cellular processes. In contrast, most cancer cells rely on aerobic glycolysis, a phenomenon termed “the Warburg effect”. They catabolize glucose to produce lactate in the reaction of pyruvate reduction catalyzed by lactate dehydrogenase (LDH), even in the presence of oxygen. Because glucose oxidation using glycolysis is highly energetically inefficient, cancer cells uptake a huge amount of glucose from the environment. This phenomenon was called “addiction to sugar” [78]. An increased activity of LDH type A (LDH-A) is characteristic for cancer cells, and its down-regulation induces oxidative stress and inhibits tumor progression [79,  80]. 

Large-scale studies have shown that in yeast 30% of genes induced by glucose starvation could be regulated by HSF [81]. Also mammalian HSF1 is engaged in the regulation of glucose metabolism. It has been concluded that HSF1 promotes glycolysis because its deficiency in MEFs leads to a reduced dependence to glucose. Additionally, such cells can better tolerate low glucose conditions and possess a lower activity of LDH [12]. Expression of LDH depends on HSF1, which binds to the *LDHA *gene promoter in human breast cancer cells overexpressing ERBB2. Down-regulation of HSF1 in these cells causes the inhibition of *LDHA* gene expression, and subsequently decreased glycolysis and cell growth retardation [82]. 

Growth factor and nutrient signaling is integrated mainly by mTOR (mammalian target of rapamycin) kinase. It regulates protein translation to coordinate growth, proliferation, and cell motility. Inhibition of mTOR kinase with rapamycin negatively influences translation, which results in the G1 cell cycle arrest and cell size reduction [83]. This effect is amplified in cells lacking HSF1. Thus, it was suggested that HSF1 could influence protein biosynthesis in a mTOR-dependent manner. In *Hsf1*-/- MEFs, a lower level of key ribosomal proteins and phosphorylated p70S6 kinase (which cooperates with mTOR) was found [12]. On the other hand, in human triple-negative breast cancer line Hs578T, HSF1 does not affect the activity of the mTOR pathway. The level of phosphorylated downstream components of the mTOR pathway (S6 ribosomal protein and 4EBP1) was not changed in these cells after *HSF1* silencing [14].

mTOR is negatively regulated by AMPK (AMP-activated protein kinase), which senses cellular energy status. Activation of AMPK was noticed in the liver of HSF1-depleted mice. It changes the metabolic balance toward the utilization of carbohydrates as an energy source, and the suppression of lipid synthesis (thus fat accumulation in *Hsf1*-/- mice is decreased). This is a consequence of enhanced insulin signaling associated with the activation of the IR/IRS-AKT pathway. Conversely, HSF1 activation promotes growth of premalignant cells and hepatocellular carcinoma development by stimulating lipid biosynthesis and perpetuating chronic hepatic metabolic disease induced by carcinogens [13]. Insulin plays a significant role in tumorigenesis [84], hence interplay between HSF1 and insulin signaling possibly is not limited to hepatic metabolism.

## HSF1 IN METASTASIS AND ANGIOGENESIS

Metastasis is a multistep process including the dissociation of cancer cells from primary sites, survival in the vascular system, and proliferation in distant target organs. Dissociated cells have to overcome anoikis and become anchorage-independent, then they are free to disseminate and colonize foreign tissues. The capacity to produce metastasis, which is the main cause of death in cancer patients, is a characteristic feature of malignant tumors [85]. 

HSF1 was found among six metastasis-promoting genes in malignant melanoma cells [26]. Association of HSF1 expression with metastatic potential was also described in prostate, hepatocellular, breast, colon, and lung tumors [17, 86, 21,  14, 16]. It was concluded that HSF1 supports anchorage-independent growth because *Hsf1*-/- MEFs fail to form colonies in soft agar (in contrast to wild type cells) [49]. Also the motility of* Hsf1*-/- MEFs, as well as bone marrow cells isolated from *Hsf1*-/- mice, is reduced in comparison to wild type cells [54,  55]. On the other hand, in mouse melanoma cells expressing constitutively active HSF1, an enhanced mobility and more dynamic anchorage-independent growth was observed [87]. Decreased expression of several genes involved in focal adhesion (*Vcl*, *Cav1*, *Capn1*) was found in such cells. Thus, it could be assumed that HSF1 promotes metastasis by facilitating cell migration. In *Hsf1*-/- MEFs, a decreased activity of RAS, MAP kinases (mainly ERK/JNK) and EGFR level was found, which suggested that HSF1 could regulate MAPK signaling pathway and EGFR expression [54]. 

Among the critical steps of cancer metastasis is the growth of a network of new blood vessels, which is called tumor angiogenesis. This process can be supported by HSF1 as well. The mean vessel area in ERBB2-induced breast cancer tumors was in *Hsf1*-/- mice almost twice as small as in wild type animals. Impairment of angiogenesis was observed also in the xenograft model using human breast cancer cells with HSF1 depletion [14]. Additionally, it has been shown that HSF1 supports the mobilization and recruitment of bone marrow-derived stem/progenitor cells in ischemia-induced angiogenesis, which contributes to neovascularization and promotes blood flow recovery [55]. Insufficient angiogenesis in *Hsf1*-/- mice is associated with the suppression of the HIF1 pathway, which is involved in tumor angiogenesis. The suppression of HIF1A in *Hsf1*-/- cells correlates with down-regulated expression of the RNA-binding protein ELAVL1 (HuR) [14]. HuR is a major regulator of translation, which promotes the translation of HIF1. Thus, *HSF1* knockdown apparently reduces the translation of *HIF1A*. 

## HSF1 AND DRUG RESISTANCE OF CANCER

Drug resistance is the major complication in chemotherapy, frequently resulting in failure of cancer treatment. There is a wide variety of molecular mechanisms involved in the resistance of cancer cells, which include: (i) increased drug efflux or decreased inward transport leading to decreased intracellular drug accumulation; (ii) increased inactivation or detoxification of drugs; (iii) decreased conversion of drug to an active form; (iv) altered quantity or activity of target proteins; (v) increased DNA repair; (vi) evasion of apoptosis. HSF1 can modify some of these pathways altering the final chemosensitivity of treated cells.

The cytoprotective and anti-apoptotic role of HSF1 is generally linked with the regulation of HSPs expression. The main role of HSPs is to maintain protein homeostasis, which enables cell survival exposition to harmful conditions [88]. However, HSPs also prevent apoptosis by direct physical interactions with apoptotic molecules. HSPs inhibit the activation of the intrinsic pathway of apoptosis by blocking the redistribution of BAX to mitochondria and the release of cytochrome c, or by preventing apoptosome formation. HSPs can also effectively inhibit the external (receptor) apoptotic pathway by inhibiting DISC (death inducing signaling complex) activity or by preventing the BID (BH3 interacting domain death agonist) pro-apoptotic factor activation after TNFα treatment [89]. The cytoprotective properties of HSPs can generate serious problems in anticancer therapy. 

Increased levels of one or more HSPs were found in a wide range of tumors and cancer cell lines [51], but their role in chemoresistance is less obvious. There are several reports showing that the up-regulation of HSP90, HSPA1 or HSPB1 following heat shock is associated with cell resistance to cisplatin or doxorubicin [90-94]. Furthermore, the damages induced by doxorubicin are more efficiently repaired following heat shock, which is correlated with nuclear translocation of HSPB1 and HSPA1 [95]. Additionally, it was reported that heat-induced carboplatin resistance of hepatoma cells is mediated by HSPA1 [96]. Nevertheless, there are also reports that the activation of HSPs expression did not enhance the survival of different cancer cells during cisplatin or colchicine, 5-fluorouracil, actinomycin D, and methotrexate treatment [90,  91,  97-99]. Moreover, diminished HSPs expression resulting from HSF1 silencing did not abrogate the resistance of HeLa cells to cisplatin [100] or MeWo cells to dacarbazine [101]. On the other hand, the down-regulation of HSPB1 expression enhances the cytotoxic effect of gemcitabine in resistant pancreatic cancer cells [102]. In MCF7 breast cancer cells selected for doxorubicin resistance an opposite effect was observed: HSF1 and HSPB1 expression was diminished. After the restoration of HSPB1 cells were more sensitive to doxorubicin [103]. Thus, the susceptibility of cells to chemotherapeutics may not simply depend on HSPs expression, but the presence of HSPs could be a secondary effect of HSF1 activity. The HSF1-dependent chemoresistance of cancer cells could be connected to its interactions with other proteins and/or its impact (direct or indirect) on expression of non-*HSPs* genes. It was shown that HSF1 is required for chemotherapy induced autophagy, since it directly up-regulates the expression of the *ATG7* gene (autophagy-related protein 7) [56] and it is necessary for the expression of sequestosome 1 (p62/SQSTM1), a protein involved in the delivery of autophagic substrates and nucleation of autophagosomes [104]. Autophagy can induce resistance to apoptosis and enhance survival under conditions of metabolic stress [105]. Modulation of autophagy by HSF1 could be also mediated by BAG3 (BCL2-associated athanogene 3). BAG3 is a mediator of macroautophagy and chaperone-assisted selective autophagy [106]. Its high expression was observed in several tumor types [107]. BAG3 is a co-chaperone protein interacting with HSPA family members, whose expression is directly regulated by HSF1 [108]. Additionally, BAG3 interacts with the anti-apoptotic BCL-2 protein family members (BCL2, Bcl-X_L_/BCL2L1, and MCL1) leading to their enhanced stability [109] and inhibition of apoptosis in cancer cells [107]. 

HSF1 can modify the resistance of cancer cells to drugs increasing their efflux by ABC transporters. There are several reports indicating that HSF1 modulates the expression of ABCB1 (known as MDR1 or P-gp), which often mediates the drug resistance. However the role of HSF1 in ABCB1 induction is not completely defined. There are reports showing that overexpression of HSF1 results in the up-regulation of *ABCB1 *and the enhanced ability to drugs efflux, which causes resistance to doxorubicin [110-112]. On the contrary, others demonstrated that lack of HSF1 could be connected with the up-regulation of* ABCB1* expression [103,  113]. Different mechanisms have been proposed for HSF1 contribution in the regulation of* ABCB1* gene expression: direct binding to the promoter resulting in either activation or inhibition of its transcription, or post-transcriptional regulation of the *ABCB1* expression. The regulation of *ABCB1* expression depends on different transcription factors, including p53 [114] and NFκB [103,  113]. Because HSF1 can interplay with both NFκB and p53 [60, 115], the relationship between HSF1 and multidrug transporters is apparently conditional and depends on cellular environment, the p53 status and specificity of the drug. 

## HSF1 AS A TARGET OF ANTICANCER THERAPY

The concept of targeting protein homeostasis in cancer cells by disrupting HSP90 (one of the most common stress-related proteins) and the proteasome was promulgated in the early 1990s. Currently, there are a few dozen active clinical trials for HSP90 inhibitors in the treatment of different types of cancer. Inhibitors of HSP40, HSP27, and HSP70 have been applied in combination with HSP90 inhibitors and other antineoplastic drugs as well [116]. However, the treatment of cancer cells with HSP or proteasome inhibitors results in the HSF1 activation and compensatory induction of HSPs, therefore reducing the antitumor activity of such inhibitors. Thus, HSF1 itself becomes an attractive target of anticancer therapy due to its involvement in different cancer-related processes. Down-regulation of HSF1 in cancer cells correlates with an elevated apoptotic index, reduced cell proliferation and tumor growth *in vivo* [12,  14]. Its down-regulation sensitized cancer cells to radiotherapy [117], to hyperthermia coupled with cisplatin treatment [100], or to HSP90 inhibitors [24,  104]. Several compounds have been tested as HSF1 inhibitors (i.e. quercetin and its derivatives, benzylidene lactam, triptolide, emunin and its derivatives), yet functional results were not satisfactory [118]. The reduction of HSF1 expression was achieved in pancreatic cancer cells by thiazole nucleoside analog [119] or in renal cancer cells treated with ritonavir (originally developed as an inhibitor of HIV protease) in combination with 17-AAG (HSP90 inhibitor) [120]. Also sulphoraphane and phenethyl isothiocyanate, a naturally occurring isothiocyanates have been shown to induce apoptosis in breast cancer cells by targeting HSF1 and HSPs [121, 122].

Several high-throughput screens for new HSF1 inhibitors have been performed, and different promising compounds were selected [123-126]. Large screen conducted by Santagata *et al*. [125] identified five diverse classes of small-molecule natural products (limonoids, curvularins, withanolides, celastraloids, and colletofragarones) bearing thiolreactive enone moieties as potent HSF1 inhibitors, which were evaluated for their anticancer activity against human glioma cells. Finally, withaferin A (WA), a naturally occurring steroidal lactone, was tested and found to be active in an orthotopic human glioma xenograft model in mice. Although WA is a promising anticancer and radio-sensitizing compound without any noticeable systemic toxicity, it is not a specific HSF1 inhibitor. It induces proteasomal degradation of BRCA1 [127], inhibits the Akt/mTOR signaling pathway [128] and modulates several other key cell-survival and regulatory pathways [129]. A more specific HSF1 inhibitor seems to be KRIBB11, which is a cell-permeable 2,6-diaminopyridine compound. It interacts with HSF1 in a reversible manner and blocks the transcription of *HSP*s. KRIBB11 exhibits antiproliferation activity against several cancer cell lines and suppresses HCT116-derived tumor growth in mice without body weight loss. This makes it an attractive candidate that could be further tested for cancer treatment in combination with other anticancer therapies [124]. Interestingly, inhibition of protein translation, e.g. by cycloheximide or rocaglate derivatives (which inhibit elongation or initiation, respectively), results in HSF1 inactivation. The most potent rocaglate derivative, named Rohinitib (or RHT), abolishes HSF1 binding throughout the genome and selectively impairs the proliferation of both malignant and premalignant cells with early-stage oncogenic lesions. This is mediated by up-regulation of TXNIP (thioredoxin-interacting protein) expression and, at a functional level, associated with reduction in glucose uptake and lactate production [126].

Since HSF1 plays a vital role in cytoprotection from high temperatures, acidosis, and neurodegenerative disorders, its therapeutic inhibition must be limited exclusively to the tumor area. Thus, treatment with HSF1 inhibitors will be possible only after minimizing disadvantageous side effects of such therapy. Therefore, it might become a promising element of anticancer treatment in the future when localized inhibition of HSF1 activity would be possible. 

## CLOSING REMARKS

HSF1 overexpression in cancer was first noticed 14 years ago [17], then its elevated expression was linked with higher malignancy, potential to metastasis, and a reduced survival rate of cancer patients. Studies on the role of HSF1 in carcinogenesis have accelerated since its potential to support neoplastic transformation has been shown [12]. The majority of data on cancer-related potential of HSF1 comes from studies on *Hsf1*-/- MEFs and mouse models of tumorigenesis. Their conclusions are schematically summarized in Fig. (**3**); some of these observations were apparently confirmed in human cancers. *Hsf1* knockout has a minimal effect on the proliferation of normal primary cells. However, a lack of HSF1 makes MEFs and mice less susceptible to neoplastic transformation [12-15,  65]. Based on these observations it was concluded that HSF1 supports cancer initiation and growth, although an exact mechanism on how it is achieved remains elusive. Elevated expression of HSPs frequently observed in cancer cells apparently contributes to specific HSF1-related features of tumor phenotype [52]. On the other hand, expression of HSPs is not exclusively dependent on HSF1, while HSF1 can also regulate numerous non-*HSPs* genes and modulate different signaling pathways by protein-protein interactions. Hence, the pro-survival function of HSF1 mediated by HSPs and other chaperones, though very important in that process, is not exclusive. Studies using *Hsf1*-/- mouse model suggest that HSF1 can modulate mTOR signaling, glucose metabolism, insulin signaling and lipid synthesis [12,  13]. An influence of HSF1 on mTOR signaling is not clear, since different results were obtained in *Hsf1*-/- MEFs and in human breast cancer cells after HSF1 silencing [12,  14]. Thus, the question of how HSF1 influence the mTOR pathway remains open. The impact of HSF1 on glucose metabolism was also confirmed by the finding that HSF1 directly activates the *LDHA* gene, which supports “addiction to sugar” in human breast cancer cells [82]. On the other hand, HSF1 inactivation (by inhibitors of translation initiation) is associated with diminished glucose uptake and lactate production, what results in reduced survival of malignant cells [126]. Additionally, HSF1 can influence signaling through RAS protein, MAP kinases, EGFR, and HIF1, modulating cell motility and angiogenesis [14,  15, 49,  54,  55]. Therefore, it was concluded that HSF1 may support migration of cancer cells and metastasis. This HSF1 action could be performed *via *HSPs, since it has been shown that HSPB1, HSPH4, and HSPB5 also promote cell migration and invasion activating the MAPK kinase/ERK pathway [52]. HSF1 definitely cooperates with p53 and NFκB signaling pathways having an impact on cell cycle, apoptosis, and DNA repair or senescence, although details of the crosstalk between these signaling pathways remain to be elucidated [60,  61, 66]. Considering its crucial role in many different processes important for malignant transformation and sensitivity of cancer cells, HSF1 appears an attractive and potent target for future anticancer strategies.

## CONFLICT OF INTEREST

The author(s) confirm that this article content has no conflicts of interest.

## Figures and Tables

**Fig. (1):**
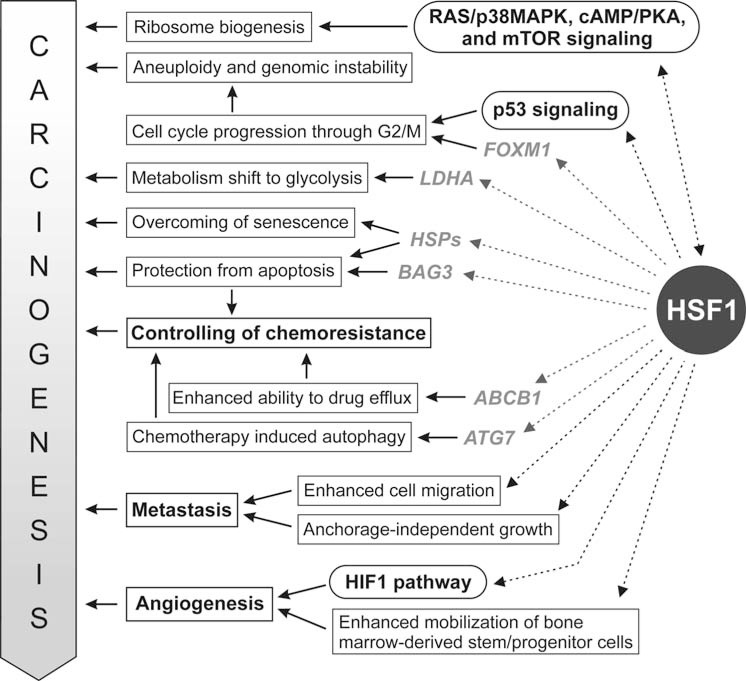
HSF1 functions that support carcinogenesis. Rectangles represent cancer-related processes effected by HSF1. Major signaling pathways influenced by HSF1 are given in rounded boxes. Genes directly regulated by HSF1 are shown in grey.

**Fig. (2):**
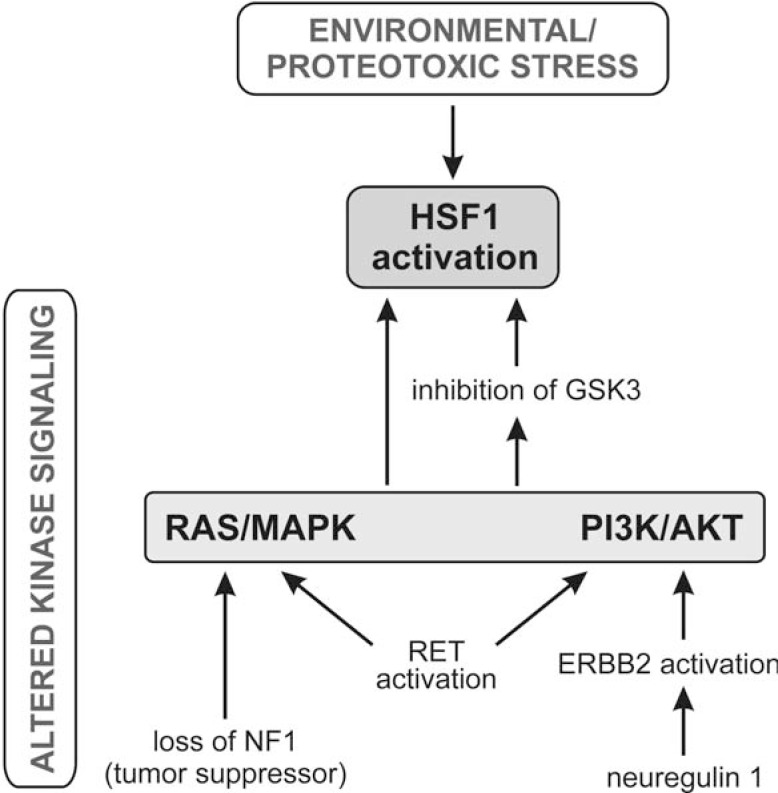
Different ways of HSF1 activation: due to proteotoxic stress or altered kinase signaling (shown are pathways documented in the literature).

**Fig. (3):**
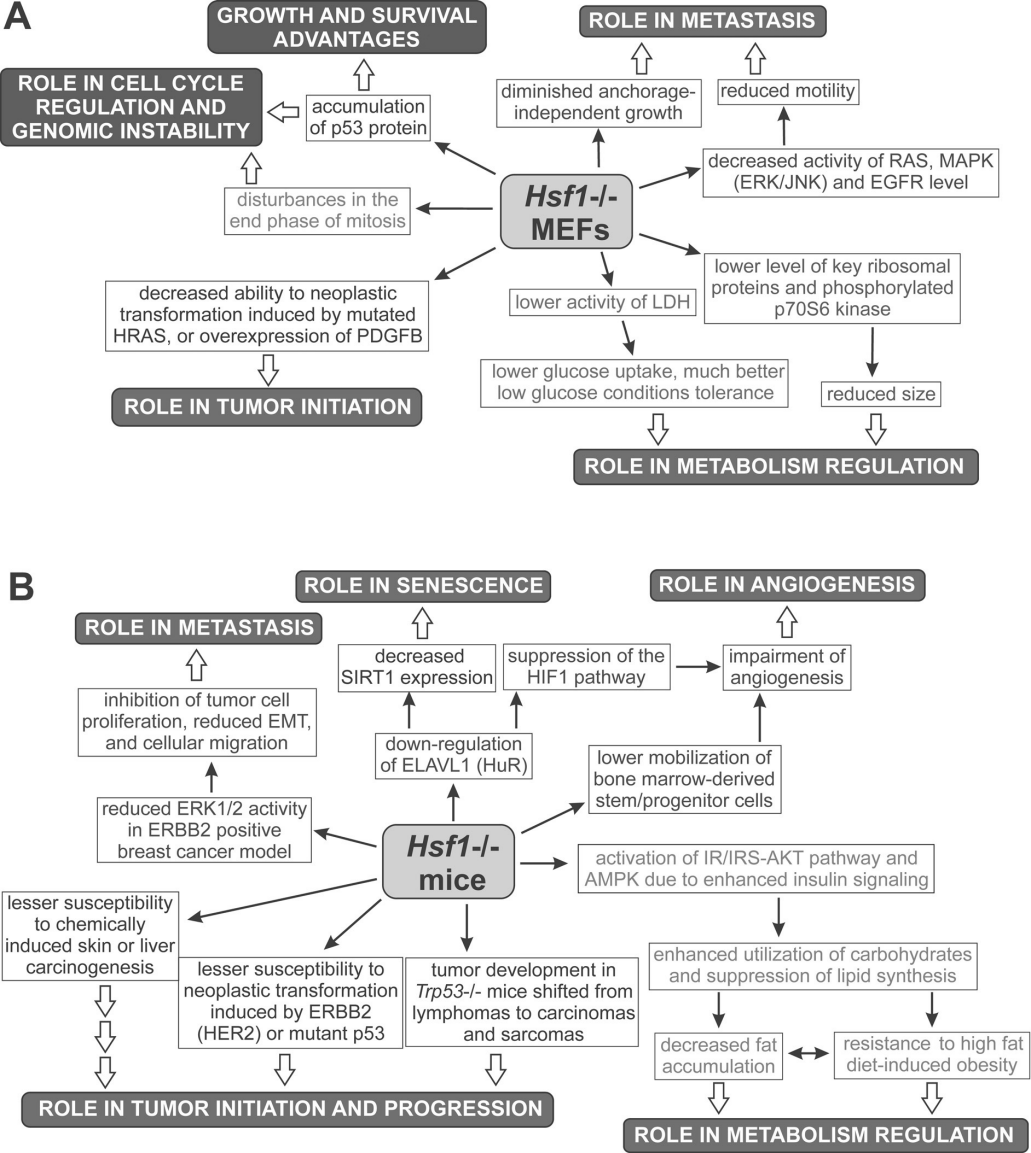
Effects of HSF1 targeting observed in mouse embryonic fibroblasts (MEFs) (A) or in mice (B). Conclusions from these observations are given in black boxes.

**Table 1:** Properties of the mammalian heat shock factor family members.

	HSF1	HSF2	HSF3	HSF4	HSF5	HSFY
**Expression**	ubiquitous	ubiquitous	ubiquitous (in mice)	tissue-specific, mainly in lens, brain, lung	tissue-specific, exclusively in testes	tissue-specific, primarily in testes
**Activation**	in response to stress (e.g. heat shock)	during development and differentiation	in response to stress	constitutive DNA-binding activity	not known	not known
**Main function**	activation of *HSP* genes, maintenance of cellular integrity during stress, and development of thermotolerance	role in oogenesis, spermatogenesis, and brain development	activation of stress-responsive genes other than *HSPs*	role in development of sensory organs (in cooperation with HSF1)	not known	potential role in spermatogenesis
**Additional functions**	role in oogenesis, spermatogenesis, brain development, immune responses, and carcinogenesis	modulation of the HSF1-mediated gene expression (possibly by formation of heterotrimers)		role in senescence and carcinogenesis		
